# The Use of Artificial Intelligence in Complementary and Alternative Medicine: A Systematic Scoping Review

**DOI:** 10.3389/fphar.2022.826044

**Published:** 2022-04-01

**Authors:** Hongmin Chu, Seunghwan Moon, Jeongsu Park, Seongjun Bak, Youme Ko, Bo-Young Youn

**Affiliations:** ^1^ Daecheong Public Health Subcenter, Incheon, South Korea; ^2^ Department of Global Public Health and Korean Medicine Management, Graduate School, Kyung Hee University, Seoul, South Korea; ^3^ Department of College of Korean Medicine, Wonkwang University, Iksan, South Korea; ^4^ National Institute for Korean Medicine Development (NIKOM), Seoul, South Korea; ^5^ Department of Preventive Medicine, College of Korean Medicine, Kyung Hee University, Seoul, South Korea

**Keywords:** artificial intelligence, complementary and alternate medicine, traditional medicine, digital health, CAM

## Abstract

**Background:** The development of artificial intelligence (AI) in the medical field has been growing rapidly. As AI models have been introduced in complementary and alternative medicine (CAM), a systematized review must be performed to understand its current status.

**Objective:** To categorize and seek the current usage of AI in CAM.

**Method:** A systematic scoping review was conducted based on the method proposed by the Joanna Briggs Institute. The three databases, PubMed, Embase, and Cochrane Library, were used to find studies regarding AI and CAM. Only English studies from 2000 were included. Studies without mentioning either AI techniques or CAM modalities were excluded along with the non-peer-reviewed studies. A broad-range search strategy was applied to locate all relevant studies.

**Results:** A total of 32 studies were identified, and three main categories were revealed: 1) acupuncture treatment, 2) tongue and lip diagnoses, and 3) herbal medicine. Other CAM modalities were music therapy, meditation, pulse diagnosis, and TCM syndromes. The majority of the studies utilized AI models to predict certain patterns and find reliable computerized models to assist physicians.

**Conclusion:** Although the results from this review have shown the potential use of AI models in CAM, future research ought to focus on verifying and validating the models by performing a large-scale clinical trial to better promote AI in CAM in the era of digital health.

## Introduction

Artificial intelligence (AI) comprehensively refers to the field of studies where cognitive functions, such as facilitating learning, reasoning, and self-correction, were conducted by machine or computer systems since John McCarthy initiated AI “to make machines behave as if they were knowledge of human behavior” at the Dartmouth conference (Ramesh et al., 2004; McCarthy et al., 2006; Ben-Israel et al., 2020). Over the last decade, the emergence of data-based medicine tendencies in the clinical environment has facilitated the convergence of medicine and AI (Krittanawong et al., 2017; Johnson et al., 2018).

As the integration of AI and medicine accelerates in various fields, healthcare and biomedical research trends are gradually changing (Yu et al., 2019; Adeluwa et al., 2021). Trained with thousands of images of diabetic retinopathy, AI achieves physician-level sensitivity and specificity in diagnosing referable diabetic retinopathy (Gulshan et al., 2016). AI also shows the possibility of a clinical decision support system of Alzheimer’s disease with training functional magnetic resonance imaging (fMRI) (Hu et al., 2016a). Likewise, the application of the AI system has expanded to diagnostics, interpretation, preventative, and prediction and support medical staff (Kundu et al., 2000; Jha and Topol., 2016). Especially, the utilization of either AI or deep learning (DL) methods on the various datasets on the drug and food, chemical compounds, or genomic biomarkers was computed and characterized for predicting risk for the prediction of drug-induced liver injury (DILI) (Adeluwa et al., 2021; Vall et al., 2021).

After the global COVID-19 pandemic, decreasing the burden on the healthcare system and reducing medical costs have emerged as an emerging issue in the healthcare system. These induce AI use in the medical field (Yu et al., 2019; Albahri et al., 2020a; Albahri et al., 2020b; Ben-Israel et al., 2020).

Furthermore, AI integration is accelerating in the conventional medical field, and the introduction of AI in complementary and alternative medicine (CAM) is also being attempted (Coleman et al., 2020; Khan et al., 2021). According to the National Health Interview Survey (NHIS) conducted in the United States, 33.2% of adults and 11.6% of children respond they use CAM, and 30% of the medical payments ($14.7 billion) were paid to CAM (Barnes et al., 2004; Barnes et al., 2008). AI is currently used for pattern diagnostics, symptoms classifications, and finding drug candidate substances in the CAM or traditional medicine fields (Zhang et al., 2020; Feng et al., 2021a; Alice et al., 2021).

In traditional medicine, AI techniques have recently been utilized to develop a prescription decision supporting system using traditional contexts or explore the efficacy of herbal extracts and prescriptions (Han et al., 2018; He et al., 2019; Zhou et al., 2021). For instance, numerous research studies were conducted to scrutinize neuroprotective compounds *via* machine learning methods from Xiaoxuming decoction, which affects regulating vascular function and treating stroke recovery (Fu et al., 2013; Shen et al., 2021; Yang et al., 2019; Zhang et al., 2021).

Although the applications of AI in CAM are expected to show various possibilities in advance of CAM, qualified and comprehensive reviews are lacking. For this reason, this study aimed to analyze the current utilization of AI in CAM to summarize the evidence and suggest improvement directions for future studies.

## Materials and Methods

This systematic scoping review used the Joanna Briggs Institute guidelines for conducting systematic scoping reviews (Levac et al., 2010; Peters et al., 2015) along with the Preferred Reporting Items for Systematic reviews and Meta-Analyzes extension for Scoping Reviews (PRISMA-ScR) checklist (Tricco et al., 2018). The checklist can be found in Supplementary Appendix S1.

### Eligibility Criteria

This review was undertaken to understand the utilization of AI in CAM modalities. All types of peer-reviewed studies, such as original articles, reviews, clinical trials, and editorials, were included.

Studies had to fall into either AI categories and defined CAM modalities. Only studies written in English were considered without any restrictions to the country. Studies published after 1 January 2000 were included as there has been a significant hike from 2000 to 2020 with regard to AI (Kaul et al., 2020). Additionally, PubMed showed about 4,300 results between 2000 and 2005 and 11,000 results between 2005 and 2010 at the time during the data extraction. Non-peer reviewed studies were excluded, as the results may be fallacious (Youn et al., 2021).

### Search Strategy

The search strategy was carried out by combining the two main concepts: AI and CAM. The terms of AI were discussed among the authors and finalized by consulting various AI data specialists and professors. The CAM modalities were chosen as defined from the Johns Hopkins medicine website (Johns Hopkins Medicine, 2021). The four authors (BY, HC, YK, SM) ran several sample searches to see if the chosen keywords were relevant to locate publications. The complete keyword search strategies are reported in Supplementary Appendix S2.

### Information Sources

According to Thielen et al. (2016), it is vital to include at least two bibliographic databases for conducting a review. Therefore, three distinct databases were used for this study: PubMed, Embase, and Cochrane Library. The final search was performed on 28 May 2021 using the databases.

### Study Selection Process

After compiling all studies to EndNote 20, duplicates and non-English articles were removed as the first step of the process. The four authors (HC, SM, JP, and SB) reviewed the rest of the studies’ titles and abstracts against the aforementioned eligibility criteria. And then, the authors met and decided the studies for which they will assess the full texts. While assessing the full texts of the studies, all references of each study were also checked for more potential studies.

A quality assessment was performed by the four authors by screening a random sample of 111 studies (10% of the overall studies) and further reviewing the screening decisions to ensure inter-rater reliability. Moreover, the two authors (BY and YK) were involved in double-screening when controversy arose before making a final decision. Four studies using fuzzy logic were excluded. We determined that fuzzy logic is a technique that converts a linguistic variable, not a deep learning technique in itself (Kharal, 2009; Al-Kasasbeh et al., 2011; Al-Kasasbeh et al., 2012; Al-Kasasbeh et al., 2013). Studies on quality evaluation of herbs or traditional medicines were excluded because it was difficult to consider the use of CAM modalities (Tian et al., 2012; Wang et al., 2012; Tan et al., 2020).

### Data Extraction Process

A data extraction table was created by the two authors (BY and SM) and completed by the four authors (HC, SM, JP, and SB) to collect the pertinent information from the final studies. The table was created in the following areas: author, country, study population, sample size, AI technique, workflow of AI model, dataset used to develop the AI model, CAM modality, and outcome.

## Results

### Study Selection and Characteristics

**FIGURE 1:**
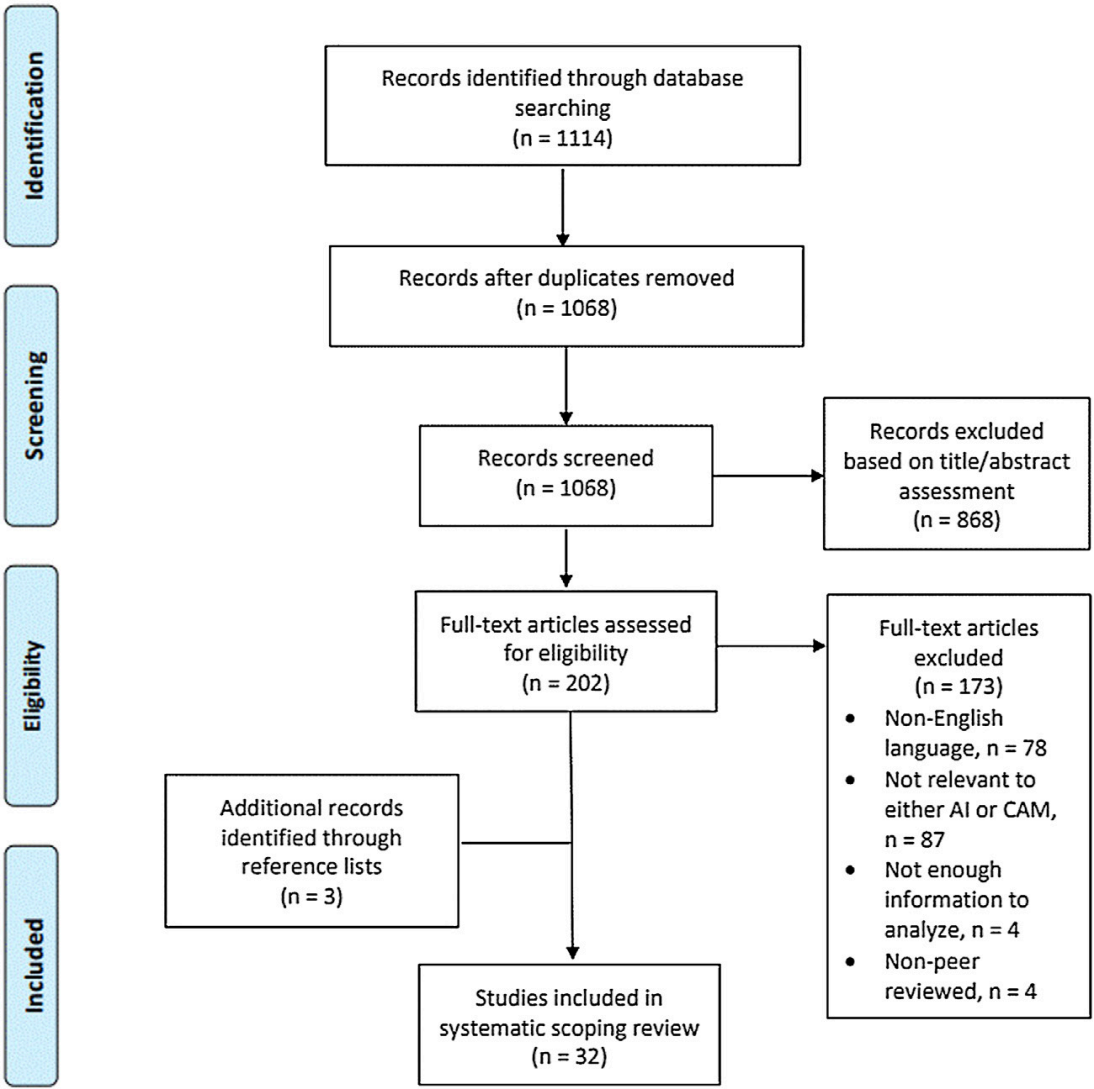
The PRISMA flow diagram for the scoping review process.

According to Figure 1, the primary search identified a total of 1,114 studies (813 PubMed, 237 Embase, and 64 Cochrane Library). A total of 46 duplicates were removed, leaving 1,068 articles for screening by titles and abstracts. After reading the titles and abstracts, 200 articles were selected for full-text assessment. During the process, 140 articles were excluded for the following reasons: 78 were written in non-English languages; 86 were not relevant to either AI or CAM; 3 had no enough information to analyze; and 4 were non-peer-reviewed. Three additional articles were found by screening references. With all that said, a total of 32 studies were finally selected.

As Table 1 shows the summary of the chosen studies, three main categories of modalities were identified: 1) acupuncture treatment, 2) tongue and lip diagnoses, and 3) herbal medicine. Other CAM modalities were combined in the fourth section. A complete set of characteristics of the included studies can be found in Table 2.

**TABLE 1:** Summary of the studies in the review.

Characteristics	Studies (n, %)
Country	
China	18, 54.54%
Taiwan	5, 15.15%
Italy	3, 9.09%
Republic of Korea	2, 6.06%
India	1, 3.03%
Hong Kong	1, 3.04%
Czech Republic	1, 3.05%
Singapore	1, 3.06%
Type of traditional medicine (n)	—
Ayurveda	1
Kampo Medicine	0
Traditional Chinese Medicine	30
Korean Medicine	2
CAM modalities	—
Acupuncture and acupoint	5, 15.15%
Herbal medicine	14, 42.42%
Tongue diagnosis	5, 15.15%
Music therapy	3, 9.09%
Symptoms pattern (Zheung)	4, 12.12%
Pulse diagnosis	1, 3.03%
Ayurveda constitution	1, 3.03%
AI technique	—
ANN	7, 17.94%
SVM	13, 33.33%
Customized or other neural networks	6, 15.38%
BP neural network	2, 5.13%
Others	11, 28.20%

**TABLE 2:** Characteristics of included studies.

CAM modality	Authors (year)	Country	Study population	AI technique	Workflow of AI model	Dataset used to develop the AI model	Main findings
Acupuncture	Jung et al. (2019)	South Korea	NA	ANN	The pattern recognition process that pertains to symptoms and diseases and informs acupuncture treatment in a clinical setting was explored. The relationship between symptom information and selected acupoints was trained using an ANN	Electronic medical records of 81 patients and a total of 232 clinical records were extracted	ANN model could predict the selected acupoints based on symptom and disease information with an average precision score of 0.865 (precision, 0.911; recall, 0.811)
Acupuncture	Han et al. (2021)	China	200 patients with peripheral facial paralysis	CNN	CNN was applied to segment images of the facial target area with facial nerve injury	200 images	Evaluating the laser speckle contrast analysis (LASCA) technology as a typing diagnosis of facial palsy in the acupuncture rehabilitation treatment
Acupuncture	Xue et al. (2011)	China	12 healthy volunteers (3 females; 9 males)	SVM	SVM classification approach to elucidate the neural response patterns	fMRI data for acupuncture GB 40 and KI 3	Group results showed distinct patterns of neural responses: predominantly positive for GB 40 and negative for KI 3
Acupuncture	Yu et al. (2019)	China	Two groups: 1) 30 healthy subjects acupuncture by TR manipulation (14 females; 16 males) and 2) 30 healthy subjects LT manipulation (12 females; 18 males)	DT, NB, SVM, KNN, LDA, LR, BP, and TSK	Multiple supervised ML classifiers were applied to quantify the modulation effects and distinguish two different acupuncture manipulations	EEG data from each subject	Classification of different acupuncture manipulations based on EEG with network features can modulate the activity of the human brain as various acupuncture manipulations have different effects on the functional brain network
Acupuncture	Yu et al. (2020)	United States	Two groups: 1) 24 patients with real acupuncture, and 2) 26 patients with sham acupuncture	DMN, SMN, SN, CEN	Used multivariate resting-state FCs to predict changes in pain severity for both real and sham treatment groups	fMRI data (30 brain regions)	Real acupuncture produced stronger treatment effects. The FCs between the mPFC and the insula, putamen, caudate, and angular gyrus were predictive of real acupuncture responses; the FCs between the mPFC and dACC, SPL, and ParaCL were predictive of sham acupuncture responses
Herbal medicine	Cao et al. (2011)	China	NA	BP neural network method	BP network model applied to PK parameters to predict morroniside	Fructus Corni was collected from Henan suppliers, and morroniside was prepared in the laboratory	BP neural network has been successfully applied to predict PK parameters of morroniside; ANN is valuable modeling that can be used to explain the relationships between the dosing regimen and PK parameters
Herbal medicine	Chen et al. (2019)	Taiwan	NA	SVM, MLR, DL, and RF	SVM and MLR methods were utilized to obtain predicted models. In particular, the DL method and RF algorithm were adopted	61,000 TCM compounds from TCM database@Taiwan	Methyl 3-*O*-feruloylquinate contained a *Phellodendron amurense* and cynanogenin A contained in *Cynanchum atratum* are capable of forming stable interactions with GSK3β
Herbal medicine	Chen et al. (2021)	China	NA	Hierarchical attentive neural network model	First stage to capture essential herbs in a prescription for its efficacy; second stage to discover essential herbal groups by mining frequent patterns	14 efficacies with their corresponding ESGHs	The hierarchical attentive neural network model is capable of capturing herbs in a prescription to its efficacy
Herbal medicine	He et al. (2019)	China	NA	QSAR model	QSAR models to predict the hepatotoxic risks of compounds by incorporating the use of 13 types of molecular fingerprints/descriptors and eight machine learning algorithms (NB, LibSVM, IBK, KStar, AdaBoostM1, Bagging, J48, and RF)	HILI dataset and CTD	By combining 13 types of molecular fingerprints/descriptors and eight machine learning algorithms, 5,416 single classifiers were developed for predicting DILI. Then, the Naive Bayes algorithm was utilized to construct a combined classifier by integrating the best single classifier of each machine learning algorithm
Herbal medicine	Jiang et al. (2016)	China	NA	SVM	From the TCM database, CNS active compounds identify and apply BBB mechanism subsequently	Three optimization parameter methods, Grid Search, GA, and PSO, were used to optimize the SVM models. Then, four optimal models were selected with excellent evaluation indexes	23 CNS active compounds collected from TCM were studied using the four discrimination models
Herbal medicine	Lin et al. (2019)	Taiwan	NA	Neural network analysis	Utilized neural network analytics to suggest prescriptions through the analysis of big data resources	261 CRC cases	Revealed 81.9% degree of similarity of CHM prescriptions and found *Atractylodes macrocephala* and *Poria* were the most commonly prescribed single herbs between the medical records and the neural network suggestions
Herbal medicine	Liu et al. (2013)	China	NA	ANN, SOM	For the pattern recognition, in order to visualize the qi- and blood-relevant compounds in a 2D space representing the structural information encoded in the molecular descriptors, the SOM was used	All chemicals of each herb were retrieved from our in-house developed database: the Traditional Chinese Medicine database and Systems Pharmacology Analysis Platform	Characterizing patterns of qi-enriching and blood-enriching herbs using deep learning methods
Herbal medicine	Elosta et al. (2006)	Czech	NA	ANN	ANN has been applied in modeling and optimization	Tanakan, *G. biloba* extract EGb 761 solution and commercial tablet of Tanakan EGb 761	The best resolution of the components was obtained within a reasonable timescale, and a new method for evaluating the quality of *G. biloba* extract was developed
Herbal medicine	Ung et al. (2007a)	Singapore	NA	PNN, kNN, SVM	PNN, kNN, and SVM were used to determine if the derived classification systems can consistently distinguish TCM herb-pairs from the non-TCM herb-pairs based on their TCM-HPs	394 TCM herb-pairs and 2,346 non-TCM herb-pairs	A 10-fold cross-validation study proved that the three AI methods could separate TCM herb pairs from non-TCM herb pairs. The accuracies for predicting TCM herb pairs are in the range of 72.1–87.9% and 91.6–97.6% for non-TCM herb pairs. The overall prediction accuracies range from 91.1 to 94.9%
Herbal medicine	Won et al. (2020)	South Korea	67 IBD patients achieved clinical remission after herbal medicine treatment	TF-IDF, DT	TF-IDF used to extract symptoms, and DC used for predicting types of pattern from presenting symptoms of patients	67 IBD patients herbal medicine prescriptions	5 patterns (large intestine type, water-dampness type, respiratory type, upper GI tract type, and coldness type) with 22 symptoms were revealed
Herbal medicine	Yang et al. (2019)	China	NA	AB, K-NN, CT, RF, NB, and molecular fingerprint descriptors	Compounds in XXMD analyzed by s-NB models were constructed based on AB, K-NN, CT, and RF algorithms and three descriptor sets; 5-fold cross-validation and test set validation were used for further evaluation	1,484 compounds from 12 herbs in XXMD from the Chinese natural product chemical composition database	RF algorithm was more applicable than others for the classification of compounds and the prediction of neuroprotective compounds against hypoxic injury and oxidative damage
Herbal medicine	Yao et al. (2019)	China	NA	ANN	Data were gathered and classified from the most famous ancient TCM book, and more than one thousand SE reports, in which two ontology-based attributions, hot and cold, are introduced to evaluate whether the prescription will cause side effects or not	242 TCM prescriptions	An ontology-based model for AI-assisted medicine side-effect prediction is proposed
Herbal medicine extraction	Li et al. (2017)	China	NA	SVM	The SVM was used to evaluate the specificity of the potential biomarkers by SVM algorithm, which was developed in the MATLAB (MATLAB R2010a, United States) kernel to map from low-dimensional to high-dimensional spaces	Cardiomyocytes divided into a control group, a periplocin low-dose group (0.2 mmol/L), and a periplocin high-dose group (0.4 mmol/L)	Identified 11 biomarkers associated with toxicity through multivariate statistical analysis. A “supervised” SVM study was used to optimize and verify the reliability of these biomarkers
Meditation (Tibetan Nyingmapa)	Lee et al. (2017)	Taiwan	30 participants (10 people with 10–30 years of experience; 10 people with 1–7 years of experience; 10 people with no experience	ANN, SVM	Applied ANN and SVM to	EEG signals from each participant	This study proposed classifier of the meditation experience
Music therapy	Raglio et al. (2020)	Italy	314 participants	DC	DC was used to predict the effect of music listening on relaxation	219 records used for the training of the classifier and 96 for testing its performance	As the overall accuracy of the DC on the test data was 0.79, the strong subjectivity of therapeutic music listening by ML techniques is an innovative approach to support music therapy practice
Music therapy	Raglio et al. (2021)	Italy	70 patients with a moderate-severe stage of dementia and behavioral disturbances	MST-based algorithm and the Auto-CM system	Computed the MST in the graph by the similarity matrix *via* Auto-CM system	27 variables (25, independent; 2, dependent) and 70 patients (Alzheimer’s *n* = 29, vascular dementia *n* = 35, mixed dementia n = 6)	The study confirmed real active music therapy interventions reduce BPSD (high scores in BI and NPI scales are predictive factors of success in the RAMT intervention) and how unsupervised ANN models can find predictive factors in clinical practice
*Prakriti* (Ayurveda constitution type)	Tiwari et al. (2017)	India	147 healthy individuals of three extreme *Prakriti* types	LASSO, elastic net, RF	Classified into one of the seven sub-types; Vata (V), Pitta (P), Kapha (K), VP, VK, PK, and VPK. Discovery set (*n* = 125) and test set (*n* = 337) were used to identify and validate biomarkers, and the validation set (*n* = 610) done by two groups of Ayurveda physicians and input these data on the deep learning discovery set (*n* = 125) and the test set (*n* = 337) were used to identify and validate biomarkers	Discovery set (*n* = 125) and test set (*n* = 337) were used to identify and validate biomarkers, validation set (*n* = 610)	Reduction in features and questions required for accurate *Prakriti* prediction. This would aid the decision-making process of *Prakriti* evaluation even by trained Ayurveda physicians
Pulse diagnosis	Tang et al. (2012)	Hong Kong	229 subjects (121 females; 108 males)	ANN, Levenberg–Marquardt	The output neurons were TCM pulse qualities operationalized as the intensity of eight elements (depth, rate, regularity, width, length, smoothness, stiffness, and strength) at six locations (left and right cun, guan, and chi)	229 samples with the eight elements at the six locations	Four-layer ANN models trained with 45 hidden neurons and the Levenberg–Marquardt algorithm performed the best
Syndromes in Chinese medicine	Xu et al. (2016)	China	835 CHD patients	REAL, ML-kNN, SVM	Using the REAL to evaluate the classification recognition accuracy for Xin qi deficiency, Xin yang deficiency, Xin yin deficiency, phlegm turbidity, and blood-stasis	Patients’ pulse data	Xin (heart) qi deficiency, Xin yang deficiency, Xin yin deficiency, blood stasis, and phlegm five-card CM diagnostic model, which had recognition rates of 80.32, 89.77, 84.93, 85.37, and 69.90%, respectively
TCM (lip diagnosis)	Li et al. (2012)	China	257 patients (132 females; 125 males)	Multi-class SVM	A multi-class SVM algorithm employed to construct the lip inspection models for diagnosis of TCM	257 lip images (90 of deep-red, 12 Pale, 62 Purple and 93 Red)	The lip diagnostic system can achieve best classification accuracy combined with SVM classifiers and SCM-REF feature selection algorithm
TCM prescription	Li H et al. (2016)	China	NA	LR, PR, SVR, ANN, and PLSR	Prediction of mechanism on the Wuji pill based on the clinical data and herbal medicine database’s framework	Herbal dataset obtained from Institute of Chinese Materia Medica at the China Academy of Chinese Medical Sciences and several clinical data	Developing simple, useful, and high-quality multi-target regression framework, which employs the correlation between targets to improve performance of learning methods, i.e., LR, PR, SVR, and ANN
TCM syndrome differentiation	Zhou et al. (2019)	China	Urine samples of 1,072 participants from nine center	SVM	The accuracy of the SVM model was used for verification. The obtained metabolomic data were input into the SVM model as a test set	Discovery set (*n* = 125) and the test set (*n* = 337) were used to identify and validate biomarkers, and the validation set (*n* = 610)	Discovered 15 CHD-PBS syndrome biomarkers and 12 CHD-QYD syndrome biomarkers, and the receiver-operator characteristic (ROC) area under the curve (AUC) values of them were 0.963 and 0.990. The established SVM model has a good diagnostic ability and can well distinguish the two syndromes of CHD with a high predicted accuracy >98.0%
Tongue diagnosis	Hu et al. (2016b)	Taiwan	51 patients, ranging between 20 and 30 years old	SVM	Prediction of the lighting condition and the corresponding color correction matrix according to the color difference of images taken with and without flash	51 tongue images	As the purpose of this study was to correct the color of tongue images under different lighting condition, the proposed automatic tongue diagnosis framework could be used applying to smartphones
Tongue diagnosis	Hu et al. (2019)	Taiwan	246 patients (54 hepatitis, 28 cirrhotic, 18 liver cancer and 146 without liver diseases)	SVM	Utilized SVM to train the tongue fur detector based on RGB values	246 tongue images	Found that some tongue features have strong correlation with the AST or ALT, which suggests the possible use of SVM-based lighting condition estimation method to provide an early warning of liver diseases
Tongue diagnosis	Pang et al. (2005)	China	525 subjects (455 patients and 70 healthy volunteers)	Bayesian networks (T-BNC, C-BNC, and J-BNC)	Bayesian network classifiers based on quantitative features, chromatic and textural measurement, are applied as the potential decision models for diagnosis	525 digital tongue images	Estimated prediction accuracy of the J-BNC is up to 75.8%. The diagnosis of four groups (healthy, pancreatitis, hypertension, and cerebral infarction) with both TPRs and PPVs higher than 75%; thus, the proposed computerized tongue diagnosis method could be used in clinical practice
Tongue diagnosis	Qi et al. (2016)	China	NA	SMOTE, SVM, RF	SMOTE was used for sample amplification; SVM and RF were applied for the analytical test and the evaluation of the classification accuracy of the model	2,230 tongue images	RF was found to give better results on the tongue color classification compared to SVM; SMOTE could improve both the whole accuracy of tongue color classification and abnormal tongue color classification
Tongue diagnosis	Zhang et al. (2019)	China	499 healthy undergraduates between 19 and 22 years old	BP neural network method	BP neural network model classifiers were designed by further calculation of the multiple fractal spectrum characteristics of digitized tongue pictures in order to classify and recognize the thin/thick or greasy characteristics of tongue coating	587 digitized tongue pictures (499 samples and 88 collected tongue pictures with obvious texture characters; 44 with a thick tongue coating and 44 with a curdy and greasy tongue coating)	Eight characteristic parameters of multiple fractal spectra of digitized tongue pictures were used as the input vectors of the three-layer BP neural network classifiers, and their coincidence rate with the judgment of TCM doctors reached 90%–93%

### Results Using Artificial Intelligence in Acupuncture Treatment

A total of five studies were related to acupuncture treatment, and most studies were regarding the selection of acupoints. It was interesting to find the Jung et al. (2019) study to predict acupoint patterns utilizing medical records based on symptom and disease information. The average precision score was relatively high, marking 0.865. Although real acupuncture treatments have been known to show more positive effects than sham acupuncture, Yeh et al.’s (2020) research confirmed using multivariate resting-state FCs.

### Results Using Artificial Intelligence in Tongue and Lip Diagnoses

Most the studies related to tongue diagnosis sought tongue color and feature classifications using AI. In the existing tongue diagnosis, color recognition, patterning, and digitization acted as limitations. However, the chosen studies have indicated classifying and quantifying patterns of color or progression through AI-applied studies. In addition, Hu et al. (2019) gave a positive outcome for the utilization of AI as preventive medicine, showing a strong correlation with the AST or ALT with some of the tongue features. The correlation of ALT with tooth marks and enlarged tongue was found; the correlation of AST with thick tongue fur and violet fur was also identified. Some notable clinical trials evaluated thickness levels compared to both ALT and AST; however, it may be difficult to evaluate on a common line as the target population was patients with chronic renal disease and liver cancer (Chen et al., 2014; Lin et al., 2015). Regardless, it can be said that the thicker tongue is close to having signs and symptoms of diseases. Because current studies that combine AI and tongue diagnosis mainly focus on tongue image extraction, further research is needed on the healthy population to find any variances.

As lip diagnosis is rarely used compared to tongue diagnosis, Li et al. (2012) attempted to construct the lip inspection model utilizing multi-class SVM algorithms and achieved a higher accuracy rate when combined with SVM and SCM-REF feature selection.

### Results Using Artificial Intelligence in Herbal Medicine

Amid the known CAM modalities, herbal medicine was the most popular modality for researchers to work with AI models. Herbal medicine studies not only compared the database of components of herbal medicine or individual herbs and existing physiological pathways to estimate the mechanism of prescription but also classified herbal medicine prescription AI models through clinical data of patients.

As there are well-developed herbal medicine databases from several countries, predicting different compounds for various diseases using AI models will assist physicians in helping patients to prevent diseases as Yang et al. (2019) found compounds against hypoxic injury and oxidative damage (Yang et al., 2019). Herbal medicine and herbal compounds included in this review are summarized in Table 3. Because the studies of Ung et al. (2007a), Ung et al. (2007b), Chen et al. (2019), Yao et al. (2019), and Jiang et al. (2016) are based on computer simulation using the established electronic database, the specific names of herbs or herbal prescriptions are not mentioned in Table 3. The case of Won et al. (2020) is not summarized in Table 3 as the study focused on finding the pattern of herbal medicine.

**TABLE 3:** Information of herbal medicine or herb compounds in the review.

Authors (year)	Modality	Species, concentration	Commercial supplier or collection cite	Reporting quality controls (Y/N)	Reporting chemical analysis (Y/N)
Cao et al. (2011)	Single herb	Frutus of *Cornus officinalis* Siebold & Zucc. (Fructus Corni)	Henan suppliers	Y, reporting the methods of preparing analytical grade acetonitrile and deionized water purification	Y, HPLC analysis, liquid chromatography mass spectrometry (LS/MC), and nuclear magnetic resonance spectroscopy (NMR)
He et al. (2019)	Single herb	Root tubers of *Polygonum multiflorum* Thunb	Not applicable	N, not applicable (computer simulation)	N, not applicable (computer simulation)
Liu et al. (2013)	Herbs	Dried roots of *Panax ginseng* C.A.Mey	Not applicable	N, not applicable (computer simulation)	N, not applicable (computer simulation)
		Radix of *Glycyrrhiza uralensis* Fisch			
		Radix of *Codonopsis pilosula* (Franch.) Nannf			
		Root of *Atractylodes ovata* Thunb			
		Rhizoma of *Dioscorea septemloba* Thunb			
		Rhizoma of *Pseudostellaria palibiniana* (Takeda) Ohwi			
		Root of *Panax quinquefolium* Linné			
		Radix of *Rehmannia glutinosa* (Gaertn.) Libosch. ex Steud			
		Root tubers of *Polygonum multiflorum* Thunb			
		Root of *Angelica sinensis* (Oliv.) Diels			
		Radix of *Paeonia japonica* (Makino) Miyabe & Takeda			
Lin et al. (2019)	Herbs	Root of *Atractylodes ovata* Thunb	Not applicable	N, not applicable (computer simulation)	N, not applicable (computer simulation)
		Sclerotium of *Wolfiporia cocos* (Schw.) Kyu. et Gilbn			
		Radix of *Astragalus membranaceus* Bunge			
		Radix of *Codonopsis pilosula* (Franch.) Nannf			
		Seed of *Coix lacrymajobi* var. mayuen (Rom.Caill.) Stapf			
		Radix of *Glycyrrhiza uralensis* Fisch			
		Pericarpium of *Citrus unshiu* S.Marcov			
		Rhizoma of *Pseudostellaria palibiniana* (Takeda) Ohwi			
		Leaf and radix of *Hedyotis diffusa* Willd			
		Rhizoma of *Pinellia ternata* (Thunb.) Breitenb			
		Radix of *Bupleurum falcatum* Linné			
		Root of *Angelica sinensis* (Oliv.) Diels			
		Root of *Salvia miltiorrhiza* Bunge			
Li Z et al. (2016)	Herbal prescription	Wuji Pills’ main herbs (Prescription of Jing yue quanshu; complete works of Zhang Jingyue; Ming Dynasty in China)	Not applicable	N, not applicable (computer simulation)	N, not applicable (computer simulation)
		Root of *Coptis japonica* Makino			
		Radix of *Paeonia japonica* (Makino) Miyabe & Takeda PALL			
		Frutus of Euodia officinalis Dode			
Yang et al. (2019)	Herbal prescriptions	Xiaoxuming Decoction (Prescription of Beiji Qianjin Yaofang; Essential Prescriptions Worth a Thousand Gold for Emergencies; Tang Dynasty in China) Fu et al. (2013)	Not applicable	N, not applicable (computer simulation)	N, not applicable (computer simulation)
Chen et al. (2019)	Herbal prescriptions	Rhizoma of *Cnidium officinale* Makino	Not applicable	N, not applicable (computer simulation)	N not applicable (computer simulation)
		Radix of *Paeonia japonica* (Makino) Miyabe & Takeda			
		Radix of *Rehmannia glutinosa* (Gaertn.) Libosch. ex Steud			
		Radix of *Spatholobus suberectus* Dunn			
		Leaf of *Morus alba* var. alba			
		Flower of *Dendranthema morifolium* (Ramat.) Tzvelev			
		Leaf of *Mentha arvensis* Linné			
		Frutus of *Tribulus terrestris* Linné			
		Frutus of *Cornus officinalis* Siebold & Zucc			
		Rhizoma and radix of *Angelica japonica* A.Gray			
		Dried roots of *Panax ginseng* C.A.Mey			
Li et al. (2017)	Single herb	Sclerotium of *Phellinus igniarius* (L. ex Fr.) Quel	Not applicable	N, not applicable (computer simulation)	N, not applicable (computer simulation)
Elosta et al. (2006)	Single herb’s extract	Extract of *Ginkgo biloba* L	IPSEN (Paris Cedex, France)	Y, reporting the methods of preparing analytical grade acetonitrile and deionized water purification	Y, electropherogram and electrophoretic separation

Although algorithm evaluation studies related to causality imputation between herbal products and herb-induced liver injury (HILI) existed, the studies were excluded because either ML (machine learning) or DL (deep learning) was not utilized (Soares et al., 2021). AI studies of the Roussel Uclaf Causality Assessment Method (RUCAM) and DILI were also exempted since HILI studies were not observed (Teschke and Danan, 2021).

### Results Using Artificial Intelligence in Other Modalities

Two studies out of the eight modalities regarded music therapy, two studies differentiated TCM syndromes, and only one study was found for each topic of meditation, pulse diagnosis, and Ayurveda constitution. With regard to the aforementioned studies, AI models were utilized for the prediction of the effectiveness of discovering the accurate models for supporting physicians while finding the right interventions for patients.

Furthermore, two review articles were found related to TCM diagnosis and TCM symptom classification (Feng et al., 2021b; Song et al., 2021). According to Feng et al. (2021b), TCM diagnosis was reviewed by classifying it into four traditional diagnostic methods: inspection, listening and smelling examination, inquiry, and palpation. This study was inadequate for further analysis as there was no specific description of which AI techniques were applied. Regarding Song et al. (2021), artificial neural network (ANN), data mining, and multivariate analysis, which are AI techniques that can be used for TCM symptoms classification, were introduced. However, the study merely introduced the possibility of using AI techniques without designating a specific symptom or diagnosis. Several studies with algorithm evaluation of pulse diagnostics existed, but these study excluded since either ML (machine learning) or DL (deep learning) methods were not utilized (Zhang et al., 2008).

## Discussion

### Main Findings

The present scoping review investigated the available evidence to identify the current use of AI models in CAM. A total of 32 studies were found for this review, and the number may seem to be small compared to the number of new techniques of AI that appear each year. However, it can be presumed that further work is recognized among CAM practitioners and researchers bridging the advanced digital tools and the principles of CAM in this digital medical age.

ML and DL, the subdomains of AI, have undergone exceptional growth in recent years. ML is fully capable of pattern recognition and training prediction models. Additionally, DL is the most recent branch of ML capable of classification and recognition tasks using a system of artificial neural networks (Tran et al., 2021). Areas where ML and DL were applied within the field of CAM were acupuncture, herbal medicine, tongue diagnosis, and pulse diagnosis. Studies have been conducted to match and learn using actual blood tests through pulse diagnosis or tongue diagnosis, which are diagnostic indicators used in traditional medication.

Recent advances in the research of herbal medicine for the prevention of coronavirus disease 2019 (COVID-19) are noteworthy, as aforementioned in the results. As Huang et al. (2020) has outlined, a number of distinctive compounds from natural products have shown the potential to inhibit SARS-COV-2 and MERS-COV, while compounds from herbal medicine have been shown to alleviate the acute respiratory infection. With the development of AI models, herbal medicine could be precisely customized per patient depending on the symptoms. According to Lynn (2019), AI can analyze more complex portions of a patients’ dataset and detect highly complex and time-dependent conditions such as adverse drug reactions and sepsis.

As the application of AI in medical fields is increasing, it aids not only in diagnostic medical devices but also in clinical trials through data amplification. In 2016, researchers at Beth Israel Deaconess Medical Center reported that an AI diagnostic program correctly identified cancer in pathology slides with 99.5% accuracy; in 2018, researchers at Massachusetts General Hospital reported that the trained AI model’s performance was as accurate as the radiologists at diagnosing intracranial hemorrhages; in 2019, researchers at Google and several medical centers reported that an AI model could detect lung cancer with the 94% of accuracy (The Harvard Gazette, 2020).

Applying AI in the various fields with CAM modalities, precise biomarker decision, measurement, and application may be required for future research. Despite these various challenges, the future of integrating AI technologies in CAM is expected to grow. In order to facilitate this, well-designed randomized controlled trials are needed to validate the AI models.

AI is applied and not limited to screening, assessment, treatment, relapse prevention, and so on in the field of CAM. In particular, the combination of CAM and AI is expected to provide better answers to the weaknesses in the existing traditional medicines. For example, AI techniques will be able to help prescribe the most effective herbal medicines because herbal medicines have a mechanism of action of complex compounds. Moreover, AI could guide making the correct diagnoses (Zhou et al., 2012; Arji et al., 2019). Assistive AI-based diagnostic models are also expected to grow in the field of decision-making model and symptoms classification using various clinical data. Further, practitioners and researchers in the field of CAM have started to build a different database using the clinical data, which can be used for drug discovery (Li et al., 2018; Zhang et al., 2018).

Concerning the study of Li A et al. (2016), the efficiency of herbal medicine was explored using the primary herb components analysis of Wuji Pill used in gynecology disease. However, neither three main herb selection criteria nor transitional bibliographic references were mentioned in detail. It is difficult to determine what prescriptions researchers used when the transitional bibliographic reference is missing in such a case.

During the search process, numerous text mining, data mining, artificial intelligence methodology development, and network pharmacology studies were recognized (Wang et al., 2009; Liu et al., 2013; Wang H. et al., 2014; Wang Y. et al., 2014; Redd et al., 2014; Liu et al., 2016; Nguyen et al., 2018; Shimada-Takaura et al., 2018; Wang et al., 2021). Even though the previously mentioned techniques deal with extensive data, the techniques do not utilize the data to either predict or learn so that the studies were excluded for further analysis.

Notably, AI is actively applied in complementary and alternative medicine. As AI is applied to drug investigation and drug efficacy estimation in the field of pharmacology, it is necessary to expand and utilize the pharmacological database in traditional medicine to be used in ethnopharmacology (Zhavoronkov et al., 2020; Gupta et al., 2021). The expansion of these databases will help drug investment not only in AI but also in fields such as network pharmacology. Importantly, predictive studies on drug precision dosing were not found in the ethnopharmacology of traditional medicine (Tou et al., 2013; Barbieri et al., 2016; Angehrn et al., 2020). Hence, topics related to AI and ethnopharmacology in traditional medicine ought to be considered for helping practitioners to prescribe personalized herbal medicines.

Nevertheless, there have been papers such as comparing the trained set with professional or physicians’ pattern diagnosis in the application of AI and CAM. However, because these datasets may involve subjectivity, studies using quantified datasets such as blood tests should be further activated in the future. Although it was excluded, not corresponding to the scope of this study, attempts to use big data such as text mining are activated in the CAM field. The application of DL seems to open new horizons for CAM research, such as a lack of participants or the discovery of hidden variability in clinical studies. However, it is necessary to continuously provide standardized official datasets or present appropriate AI models.

### Limitations of the Study

This review has several limitations to note. First of all, articles published in English were only included. It might have been better to search Chinese databases as China has a long-lasting history of using CAM modalities for many years as Traditional Chinese Medicine (Fan et al., 2018). Second, there is also a limitation in that this study does not include studies from Chinese databases. TCM encompasses a wide range of practices and is vibrantly applied throughout China; it may have been better to review TCM-related AI applications in Chinese literature.

Moreover, the eligibility criteria were defined rather broadly, including all AI techniques and CAM modalities, being the first review study to seek any potential use of AI in CAM. As this study is expected to serve as a basis for future studies, it may be meaningful to focus on using merely one AI technique per CAM intervention when there is enough information in the near future.

## Conclusion

The use of AI models has been bringing efficient solutions to a vast number of real-world clinical problems. The results from this systematic scoping review have revealed the use of AI in various CAM modalities. Even though research for AI models in CAM is at its initial stage, the results from the chosen studies are promising that the AI techniques could well be applied to assist practitioners in better serving the patients. However, applying various AI models to a wide array of CAM modalities is much needed going forward.

More importantly, in order to widely spread the utilization of AI models in CAM, concrete evidence is much needed as novel technologies may fail to be adopted without sufficient evidence. That being said, for researchers who plan to conduct research using AI methods with CAM, it is significantly vital to verify the generalizability and validate the effectiveness and the reliability for the betterment of the promotion of CAM globally.

## Data Availability

The original contributions presented in the study are included in the article/Supplementary Material, further inquiries can be directed to the corresponding author.
