# Supplementary material for: The Use of Artificial Intelligence in Complementary and Alternative Medicine: A Systematic Scoping Review
Source: Front Pharmacol. 2022 Apr 1;13:826044. doi: 10.3389/fphar.2022.826044 (PMC9011141; doi:10.3389/fphar.2022.826044)
Supplement: Supplementary file 1 [file Table1.DOCX]

SVM- support vector machine

MST- minimum spanning tree

Auto-CM- Auto-Contractive Map

NPI- Neuropsychiatric Inventory

ANN- artificial neural network

ESHGs-Efficacy-Specific Herbal Groups

MLR- multiple linear regression

DL- deep learning

RF- random forest

TCM- traditional Chinese medicine

BP- back-propagation

PK- pharmacokinetic

SVM-RFE- Support Vector Machine with recursive feature elimination

CRC- colorectal cancer

CHM- Chinese herbal medicine

EEG- electroencephalography

GB40- gall bladder 40; Acupoint name Qiuxu

KI 3- kidney 3; Acupoint name Tai Xi

fMRI- Functional magnetic resonance imaging

DT- decision tree

NB- naïve Bayes

KNN- k-nearest neighbor

LDA- linear discriminant analysis

PLSR- logistic regression

TSK- Takagi- Sugeno-Kang fuzzy system

RGB- Red green blue

AST- aspartate transaminase

ALT- alanine transaminase

TF-IDF- term frequency-inverse document frequency

DT- digital transformation

IBD- inflammatory bowel disease

GI- Gastrointestinal

DMN- default mode network

SMN- sensorimotor network

SN- salience network

CEN- central executive network

FC- functional connectivity

mPFC- medial prefrontal cortex

dACC- dorsal anterior cingulate cortex

SPL- superior parietal lobe

ParaCL- paracentral lobe

AB- Adaboost

K-NN- 𝑘 nearest neighbors

CT- classification tree

XXMD- Xiaoxuming decoction

PNN- Probabilistic neural network

HPs- herbal properties

CNN- convolutional neural networks

BNC- Bayesian network classifier

T-BNC- texture BNC

C-BNC- color BNC

J-BNC- joint BNC

TPR- true positive rate

PPV- positive predictive values

ML- machine learning

ARM- association rule mining

SMOTE- synthetic minority oversampling technique

QSAR- Quantitative structure–activity relationship

IBK- Instance Based Learner

HILI- herb-induced liver injury

CTD- Comparative Toxicogenomics Database

DILI- drug-induced liver injuries

CNS- central nervous system

BBB- blood–brain barrier

GA- Genetic Algorithm

PSO- Particle Swarm Optimization

SOM- self-organizing map

CHD- Coronary heart disease

PBS- phlegm and blood stasis

QYD- Qi and Yin deficiency
