# Supplementary material for: The Use of Artificial Intelligence in Complementary and Alternative Medicine: A Systematic Scoping Review
Source: Front Pharmacol. 2022 Apr 1;13:826044. doi: 10.3389/fphar.2022.826044 (PMC9011141; doi:10.3389/fphar.2022.826044)
Supplement: Supplementary file 2 [file Table2.DOCX]

***Supplementary Material***

**Appendix 2**. Database search strategy

1. The details of the PubMed search

("complementary therapies"[MeSH Terms] OR "complementary therapy"[Title/Abstract] OR "complementary medicine"[Title/Abstract] OR "alternative therapy"[Title/Abstract] OR "alternative medicine"[Title/Abstract]) AND ("artificial intelligence"[MeSH Terms] OR "natural language processing"[MeSH Terms] OR "deep learning"[MeSH Terms] OR "machine learning"[MeSH Terms] OR "supervised machine learning"[MeSH Terms] OR "neural networks, computer"[MeSH Terms] OR "support vector machine"[MeSH Terms] OR "computer heuristics"[MeSH Terms] OR "artificial intelligence"[Title] OR "computational intelligence"[Title] OR "deep learning"[Title] OR "machine learning"[Title] OR "supervised machine learning"[Title] OR "neural network"[Title] OR "artificial neural network"[Title] OR "convolutional neural network"[Title] OR "natural language processing"[Title] OR "computer vision"[Title]) Filters: from 2000/1/1 - 2021/12/312. Gray literature

2. The details of the Embase

1. 'alternative medicine'/exp OR 'complementary therapy':ab,ti OR 'complementary medicine':ab,ti OR 'alternative therapy':ab,ti OR 'alternative medicine':ab,ti

2. 'artificial intelligence'/exp OR 'natural language processing'/exp OR 'deep learning'/exp OR 'machine learning'/exp OR 'supervised machine learning'/exp OR 'neural networks, computer'/exp OR 'support vector machine'/exp OR 'computer heuristics'/exp

3. 'artificial intelligence':ti OR 'computational intelligence':ti OR 'deep learning':ti OR 'machine learning':ti OR 'supervised machine learning':ti OR 'neural network':ti OR 'artificial neural network':ti OR 'convolutional neural network':ti OR 'natural language processing':ti OR 'computer vision':ti

4. #2 OR #3

5. (#1 AND #4) AND (2001:py OR 2005:py OR 2007:py OR 2008:py OR 2009:py OR 2010:py OR 2011:py OR 2012:py OR 2013:py OR 2014:py OR 2015:py OR 2016:py OR 2017:py OR 2018:py OR 2019:py OR 2020:py OR 2021:py)

3. The details of the Cochrane

1. MeSH descriptor: [Complementary Therapies] explode all trees

2. ('complementary therapy'):ti,ab,kw OR ('complementary medicine'):ti,ab,kw OR ('alternative therapy'):ti,ab,kw OR ('alternative medicine'):ti,ab,kw

3. #1 OR #2

4. MeSH descriptor: [Artificial Intelligence] explode all trees

5. MeSH descriptor: [Natural Language Processing] explode all trees

6. MeSH descriptor: [Deep Learning] explode all trees

7. MeSH descriptor: [Machine Learning] explode all trees

8. MeSH descriptor: [Supervised Machine Learning] explode all trees

9. MeSH descriptor: [Neural Networks, Computer] explode all trees

10. MeSH descriptor: [Support Vector Machine] explode all trees

11. MeSH descriptor: [Computer Heuristics] explode all trees

12. #4 OR #5 OR #6 OR #7 OR #8 OR #9 OR #10 OR #11

13. ('supervised machine learning'):ti OR ('machine learning'):ti OR ('artificial intelligence'):ti OR ('computational intelligence'):ti OR ('deep learning'):ti (Word variations have been searched)

14. ('neural network'):ti OR ('artificial neural network'):ti OR ('convolutional neural network'):ti OR ('natural language processing'):ti OR ('computer vision'):ti (Word variations have been searched)

15. #13 OR #14

16. #12 OR #15

17. #3 AND #16
